# Supplementary material for: Optimisation of surfactin yield in Bacillus using data-efficient active learning and high-throughput mass spectrometry
Source: Comput Struct Biotechnol J. 2024 Feb 15;23:1226–33. doi: 10.1016/j.csbj.2024.02.012 (PMC10973723; doi:10.1016/j.csbj.2024.02.012)
Supplement: Supplementary file 1 — Supplementary material [file mmc1.docx]

**Supplementary Figures**


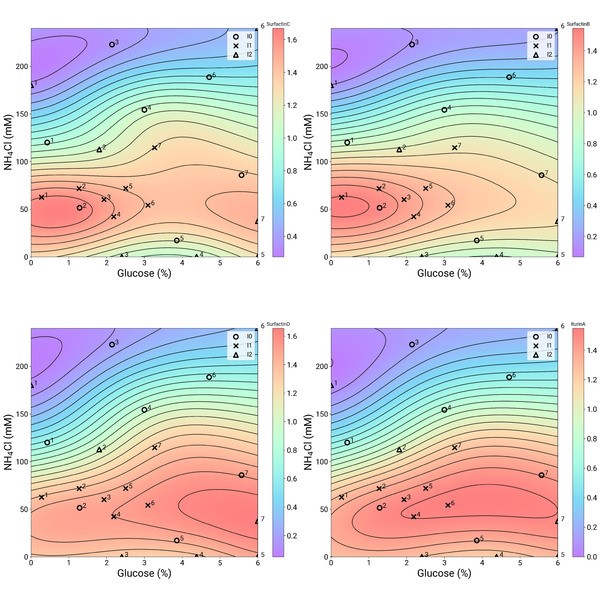


**Figure S1. Surfaces for the 4 lipopeptides that were simultaneously measures using the flow injection MS method.** Samples from all iterations are depicted in the surfaces. Colour indicates predicted titre by the Gaussian process regression model.


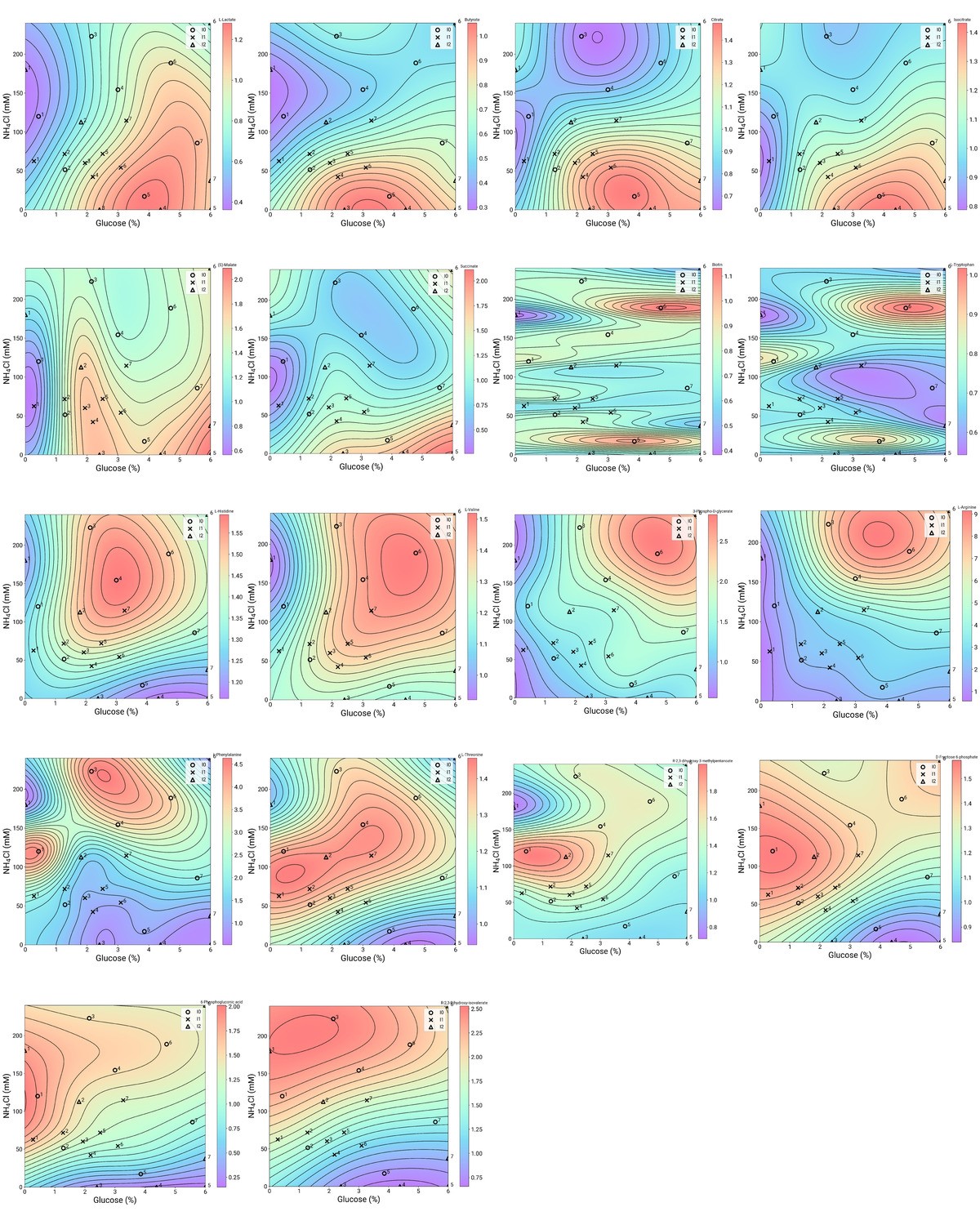


**Figure S2. Surfaces for 18 carbon/TCA-related compounds that were measured outloop using the flow injection MS method.** Samples from all iterations are depicted in the surfaces. Colour indicates predicted titre by the Gaussian process regression model.


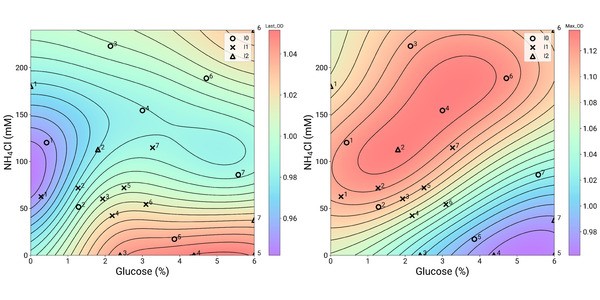


**Figure S3. Surfaces for 2 growth measurements that were obtained from the plate reader experiment.** Samples from all iterations are depicted in the surfaces. Colour indicates predicted titre by the Gaussian process regression model.


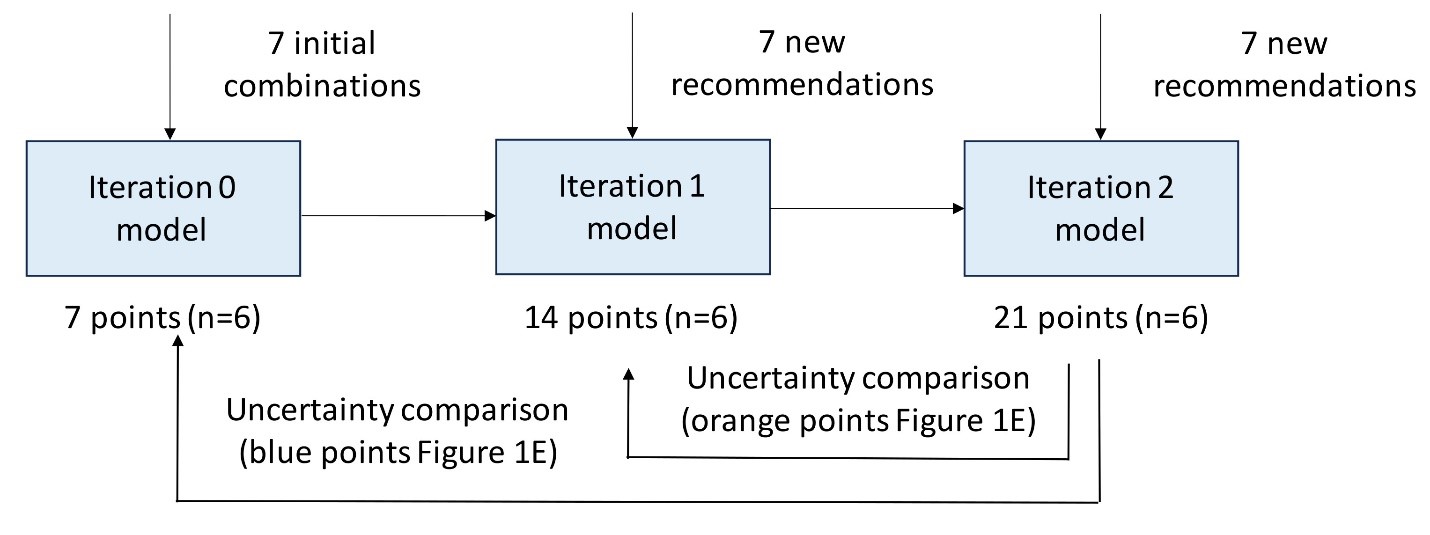


**Figure S4. Diagram showing what data serves as input to the model in each iteration and how the predicted uncertainty in the model is compared.** The model is being updated after each iteration, augmenting the available information and therefore reducing the predicted uncertainty for simulated points in the models.


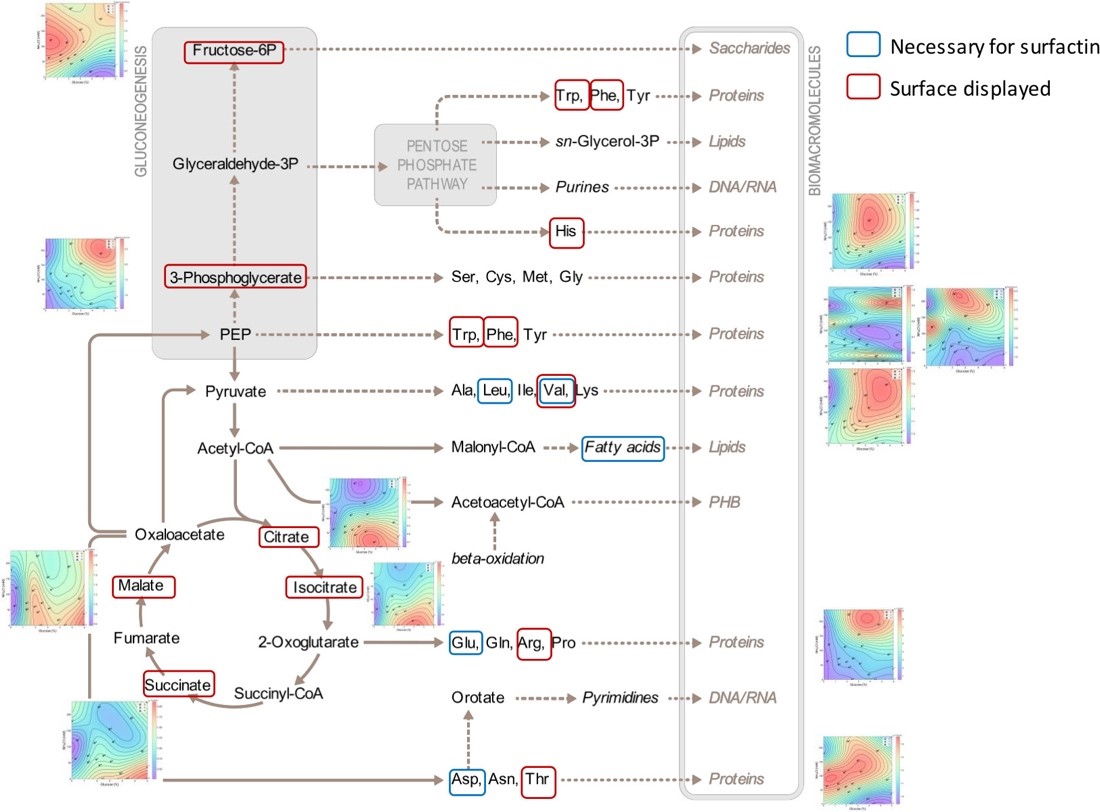


**Figure S5. Simplified pathway diagram of anabolism in *B. subtilis***, **with production surfaces assigned to the measured metabolites.** The diagram was extracted from MetaboMaps (Koblitz et al., 2020). Metabolites with available surface are enclosed with a blue box, while metabolites involved in further surfactin production are enclosed with a red box.


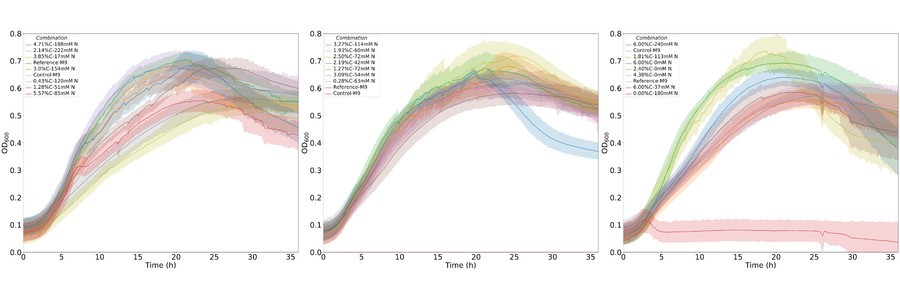


**Figure S6. Growth curves for the microplates.** From left to right, it is depicted the growth curves for different C/N combinations in Iteration 0, Iteration 1 and Iteration 2. The trend colour is given by a specific carbon and nitrogen concentration, as shown in the legend. The shadow in each trend corresponds to the confidence band calculated from the 6 biological replicates.

**
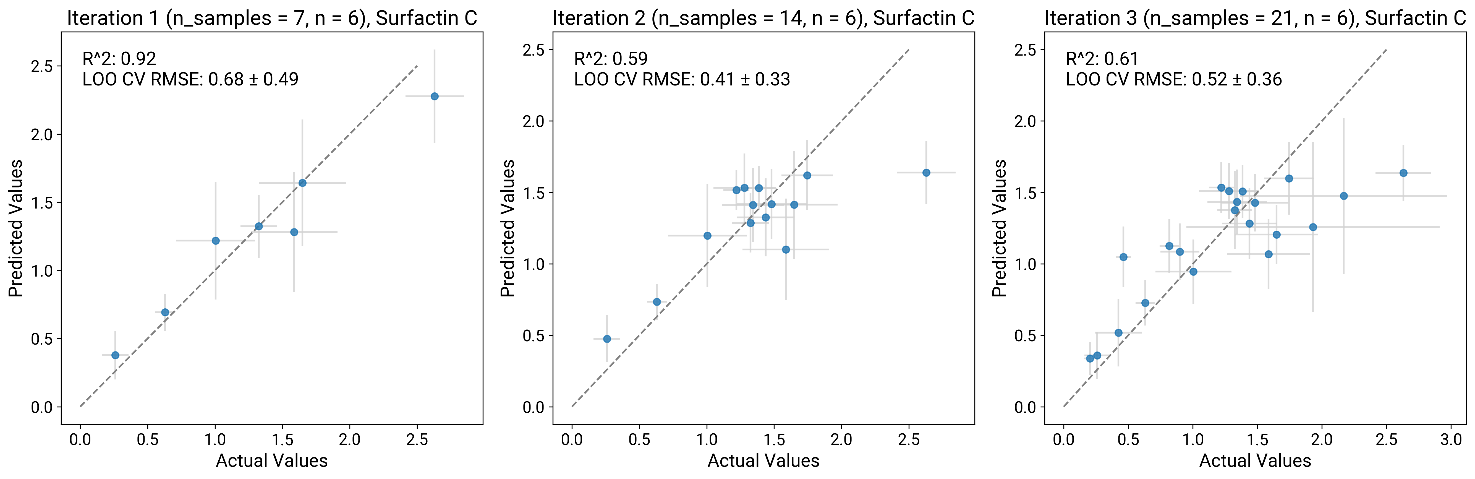
**

**Figure S7. Predicted values vs actual values plot for the Surfactin C model, per iteration.** Each point is shown with corresponding errors bars given by the replicate samples, in the case of the actual values, and the variance in the prediction of the model, for the predicted values. R^2^ scores and leave-one-out cross-validation root of the mean squared error were calculated to analyse goodness-of-fit and generalisation property of the models.

**Supplementary Tables**

**Table S1. Parameters used on the triple quadrupole mass spectrometer (QqQ-MS) runs using the developed flow injection method.**

| **Parameter** | **Value** |
| --- | --- |
| **Spray Voltage** | **Static** |
| **Positive Ion (V)** | **3500** |
| **Negative Ion (V)** | **3500** |
| **Sheath Gas (Arb)** | **35** |
| **Aux Gas (Arb)** | **5** |
| **Sweep Gas (Arb)** | **0** |
| **Ion transfer Tube Temp (°C)** | **325** |
| **Vaporizer Temperature (°C)** | **275** |

**Table S2. Precursor and product masses used for selected reaction monitoring (SRM) in the QqQ-MS**

| **Molecule** | **Polarity** | **Precursor mass (m/z)** | **Product mass (m/z)** | **Collision Energy (V)** | **Dwell time (ms)** |
| --- | --- | --- | --- | --- | --- |
| **Lipopeptides** | | | | | |
| SurfactinB | Positive | 1008.2 | 685 | 20 |  |
| SurfactinC | Positive | 1022.3 | 685 | 20 |  |
| IturinA | Positive | 1044.3 | 391 | 35 |  |
| SurfactinD | Positive | 1058.2 | 685 | 20 |  |
| **Central metabolism** | | | | | |
| L-Valine | Positive | 118.086 | 57.054 | 29.94 | 6.581 |
| L-Valine | Positive | 118.086 | 72 | 11.49 | 6.581 |
| L-Threonine | Positive | 120.066 | 74 | 11.4 | 6.581 |
| L-Threonine | Positive | 120.066 | 103 | 18.44 | 6.581 |
| L-Histidine | Positive | 156.077 | 93 | 23.62 | 6.581 |
| L-Histidine | Positive | 156.077 | 110.054 | 15.19 | 6.581 |
| L-Phenylalanine | Positive | 166.086 | 77 | 39.84 | 6.581 |
| L-Phenylalanine | Positive | 166.086 | 120.054 | 14.06 | 6.581 |
| L-Arginine | Positive | 175.119 | 70.071 | 23.7 | 6.581 |
| L-Arginine | Positive | 175.119 | 158.054 | 12.71 | 6.581 |
| L-Tryptophan | Positive | 205.097 | 146.071 | 18.18 | 6.581 |
| L-Tryptophan | Positive | 205.097 | 188.071 | 10.35 | 6.581 |
| L-Lactate | Negative | 89.024 | 45.125 | 12.2 | 6.581 |
| L-Lactate | Negative | 89.024 | 71.012 | 9.21 | 6.581 |
| Butyrate | Negative | 89.1 | 43.1 | 14 | 6.581 |
| Succinate | Negative | 117.019 | 73.113 | 11.74 | 6.581 |
| Succinate | Negative | 117.019 | 98.827 | 7.86 | 6.581 |
| (S)-Malate | Negative | 133.014 | 70.988 | 13.34 | 6.581 |
| (S)-Malate | Negative | 133.014 | 114.929 | 11.15 | 6.581 |
| R-2,3-Dihydroxy-isovalerate | Negative | 133.051 | 133.051 | 0 | 6.581 |
| R-2,3-dihydroxy-3-methylpentanoate | Negative | 147.066 | 147.066 | 0 | 6.581 |
| 3-Phospho-D-glycerate | Negative | 184.986 | 123.107 | 17.01 | 6.581 |
| 3-Phospho-D-glycerate | Negative | 184.986 | 166.917 | 9.04 | 6.581 |
| Citrate | Negative | 191.02 | 86.845 | 17.89 | 6.581 |
| Isocitrate | Negative | 191.02 | 116.958 | 14.81 | 6.581 |
| Isocitrate | Negative | 191.02 | 172.929 | 7.86 | 6.581 |
| Biotin | Negative | 245.1 | 227 | 14 | 6.581 |
| D-Fructose 6-phosphate | Negative | 259.022 | 138.929 | 16.5 | 6.581 |
| D-Fructose 6-phosphate | Negative | 259.022 | 168.708 | 8.71 | 6.581 |
| 6-Phosphogluconic acid | Negative | 275.017 | 195.143 | 21.68 | 6.581 |
| 6-Phosphogluconic acid | Negative | 275.017 | 256.899 | 12.83 | 6.581 |
